# pH as a potential therapeutic target to improve temozolomide antitumor efficacy : A mechanistic modeling study

**DOI:** 10.1002/prp2.454

**Published:** 2019-01-28

**Authors:** Angélique Stéphanou, Annabelle Ballesta

**Affiliations:** ^1^ Université Grenoble Alpes CNRS TIMC‐IMAG/DyCTIM2 Grenoble France; ^2^ INSERM and Paris Sud university UMRS 935 Team “Cancer Chronotherapy and Postoperative Liver Functions” Villejuif France; ^3^ University of Warwick Coventry UK

**Keywords:** glioblastoma, mathematical modeling, pH, pharmacokinetics‐pharmacodynamics, temozolomide

## Abstract

Despite intensive treatments including temozolomide (TMZ) administration, glioblastoma patient prognosis remains dismal and innovative therapeutic strategies are urgently needed. A systems pharmacology approach was undertaken to investigate TMZ pharmacokinetics‐pharmacodynamics (PK‐PD) incorporating the effect of local pH, tumor spatial configuration and micro‐environment. A hybrid mathematical framework was designed coupling ordinary differential equations describing the intracellular reactions, with a spatial cellular automaton to individualize the cells. A differential drug impact on tumor and healthy cells at constant extracellular pH was computationally demonstrated as TMZ‐induced DNA damage was larger in tumor cells as compared to normal cells due to less acidic intracellular pH in cancer cells. Optimality of TMZ efficacy defined as maximum difference between damage in tumor and healthy cells was reached for extracellular pH between 6.8 and 7.5. Next, TMZ PK‐PD in a solid tumor was demonstrated to highly depend on its spatial configuration as spread cancer cells or fragmented tumors presented higher TMZ‐induced damage as compared to compact tumor spheroid. Simulations highlighted that smaller tumors were less acidic than bigger ones allowing for faster TMZ activation and their closer distance to blood capillaries allowed for better drug penetration. For model parameters corresponding to U87 glioma cells, inter‐cell variability in TMZ uptake play no role regarding the mean drug‐induced damage in the whole cell population whereas this quantity was increased by inter‐cell variability in TMZ efflux which was thus a disadvantage in terms of drug resistance. Overall, this study revealed pH as a new potential target to significantly improve TMZ antitumor efficacy.

**Abbreviations**
ABC: ATP‐Binding Cassette
AIC: 4‐amino‐5‐imidazole‐carboxamide
CA: Cellular Automaton
MTIC: 5‐(3‐methyltriazen‐1‐yl)imidazole‐4‐carboxamide
ODE: Ordinary Differential Equation
PDE: Partial Differential Equation
TMZ: temozolomide

## 1 INTRODUCTION

Glioblastoma (GBM) is the most frequent and aggressive primary brain tumor in adults. It is associated with a dismal median patient survival of approximately 18 months despite intensive treatment involving surgery, radiation, and chemotherapy mainly based on the alkylating agent temozolomide (TMZ).^1^ No major therapeutic advance has been accomplished since this current standard of care was established more than 10 years ago. Moreover, this treatment is associated with moderate to severe toxicity events, which can be life threatening in some cases.^2^ Hence, innovative therapeutic strategies are urgently needed and there is scope for great progress in terms of patients survival and quality of life. TMZ is the cornerstone of GBM management but has also been approved for the treatment of other solid tumors including pituitary tumors.^3^

TMZ is a prodrug that spontaneously converts into its metabolite 5‐(3‐methyltriazen‐1‐yl)imidazole‐ 4‐carboxamide (MTIC), which is subsequently degraded into 4‐amino‐5‐imidazole‐carboxamide (AIC)–an inactive metabolite and a methyldiazonium cation, the DNA‐methylating species. The methyldiazonium cation creates DNA adducts–a marker of TMZ pharmacodynamics (PD)–that trigger DNA damage responses and potentially induce cell death.^4^, ^5^ Both TMZ and MTIC degradation rates are highly pH‐dependent as they exponentially increase and decrease with pH values, respectively.^6^

Healthy and tumor cells present different regulations of extra‐ and intracellular pH values which may influence TMZ PK although this has not been studied mechanistically up to our knowledge. Cancer cells can acidify their micro‐environment which may favor the development of resistant clones, promote tumor invasion and suppress the antitumor immune response.^7^, ^8^, ^9^ Furthermore, cancer cells may present an abnormal regulation of their intracellular pH which allow them to evade from acid‐mediated toxicities whereas healthy cells would not survive in acidic environment.^10^, ^11^ Several anticancer strategies currently under development rely on targeting the tumor pH such as the administration of proton pump inhibitors to invert extracellular/intracellular pH gradient or the design of pH‐controlled nanoparticles releasing the active compound at acid pH.^10^

Mathematical modeling of tumor acidity is not new^12^ and the integration of the intracellular pH regulation of tumor cell has also been considered a while ago^13^ by incorporating the effects of the different membrane transporters. The complex tumor cell metabolism and its evolution from aerobic to glycolysis were also considered in tumor models^14^, ^15^, ^16^ to establish the extracellular pH dynamics accompanying the tumor evolution. More recently cellular automaton approaches were developed so as to integrate the pH as an environmental constraint.^17^ Such more general and often multiscale hybrid models have now proved very useful to further evaluate consequences of treatments.^18^

We here intend to investigate TMZ pH‐dependent pharmacokinetics (PK) and simplified pharamcodynamics (PD) in solid tumors through such hybrid mathematical modeling and validate the potential of pH as a therapeutic target to increase TMZ exposure benefit both in terms of efficacy and tolerability. We build on a previously published non‐spatial model of TMZ PK‐PD which has been incorporated into a spatial hybrid framework to analyze TMZ efficacy in a space‐ and pH‐dependent manner.^4^, ^19^

## 2 MATERIALS AND METHODS

### 2.1 Non‐spatial model of TMZ cellular PK‐PD

**Figure 1:**
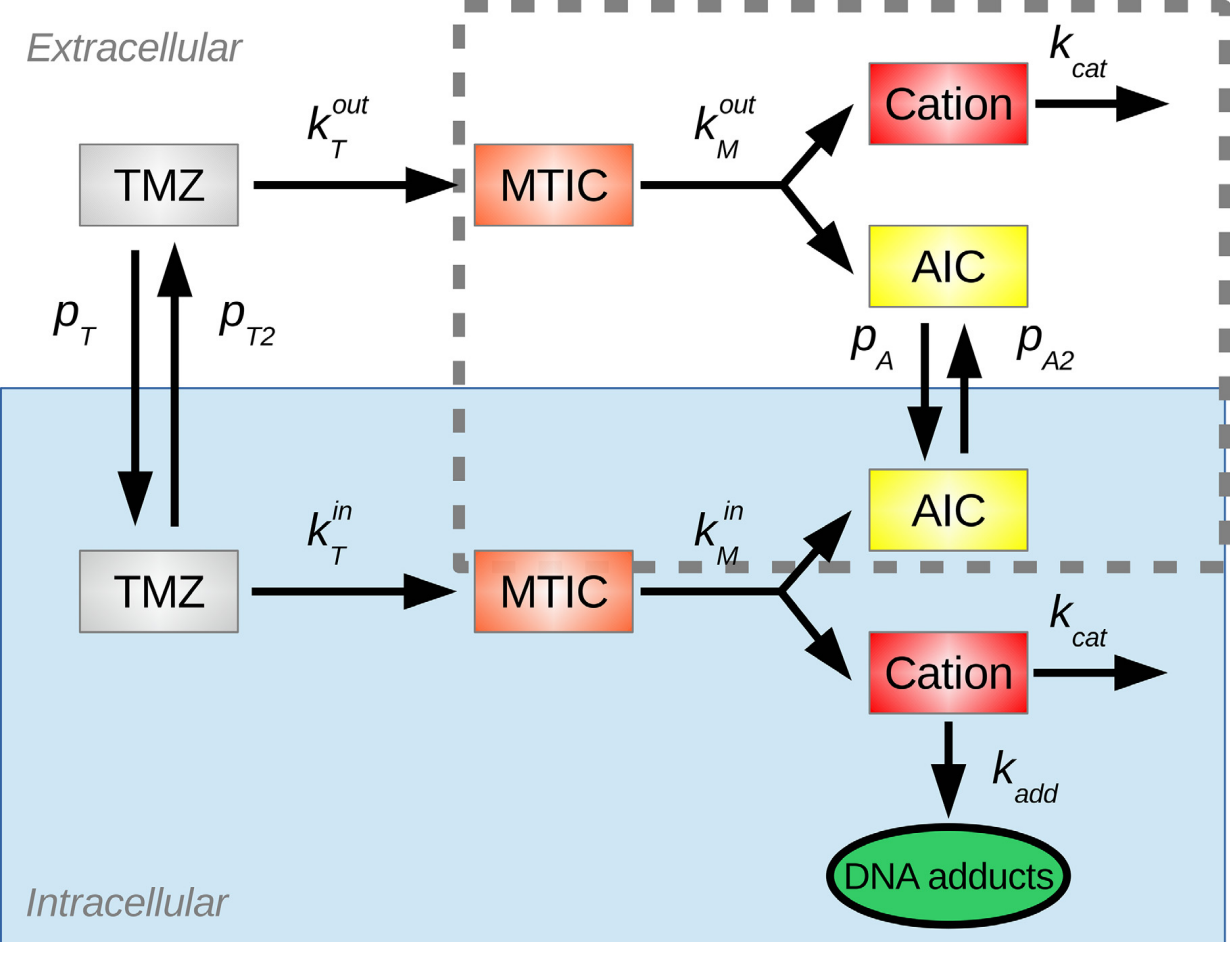
TMZ PK as considered in the original ODE‐based model. It differentiates the extra and intracellular TMZ transformation. In the integration with the cellular automaton, the elements in the dotted box are not described since they play no part in the generation of DNA adducts, the output variable of interest

TMZ cellular PK‐PD was firstly represented by an Ordinary Differential Equations (ODE)‐based model.^4^ This model considers both an extra‐ and an intracellular compartment (Figure 1). In both compartments, TMZ pH‐dependent activation into MTIC and MTIC subsequent degradation into AIC are represented by the law of mass action. MTIC dissociation produces a methyldiazonium cation that can create DNA adducts, which is also represented by the law of mass action. Because TMZ is highly lipophilic and constitutes a poor substrate of ATP‐Binding Cassette (ABC) transporters, its cellular transport is modeled as passive diffusion using Ficks first law. As MTIC displays limited ability to cross cell membranes and as the methyldiazonium cation is a highly reactive species, their transport between the extra‐ and intracellular compartments were not considered. Regarding TMZ PD, the methyldiazonium cation is the sole species able to form DNA adducts that are considered as an early marker of TMZ efficacy.

**Figure 2:**
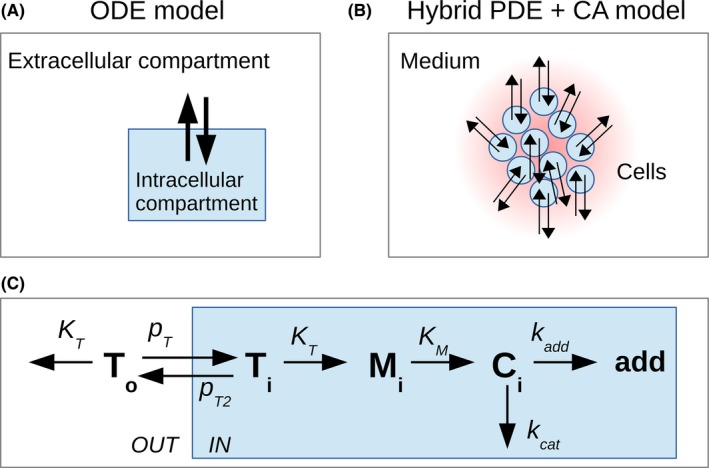
Models representations. (A) Standard two‐compartment model where the volume of the intracellular compartment is the total volume of all cells; (B) CA model where each cell is explicitly represented and its PK is individually calculated. A hybrid PDE describes the extracellular TMZ concentration in space resulting from TMZ diffusion and local exchange with cells; (C) PK model reduced to the intracellular compartment, as used in (A) and (B)

The system of ODEs for TMZ extracellular concentration ($T_{o}$) and for the intracellular dynamics of TMZ ($T_{i}$), MTIC ($M_{i}$), Cation ($C_{i}$) and DNA adducts (Add) concentrations (Figure 2) are defined by:

(1) $$\frac{dT_{o}}{dt}=\left(\frac{p_{T2}}{V_{o}}+k_{T}\left(pH_{e}\right)\right)T_{i}-\frac{p_{T}}{V_{o}}T_{o}$$

(2) $$\frac{dT_{i}}{dt}=-\left(\frac{p_{T2}}{V_{i}}+k_{T}\left({\text{pH}}_{i}\right)\right)T_{i}+\frac{p_{T}}{V_{i}}T_{o}$$

(3) $$\frac{dM_{i}}{dt}=k_{T}\left({\text{pH}}_{i}\right)T_{i}-k_{M}\left({\text{pH}}_{i}\right)M_{i}$$

(4) $$\frac{dC_{i}}{dt}=k_{M}\left({\text{pH}}_{i}\right)M_{i}-\left(k_{\mathrm{cat}}+k_{\mathrm{add}}\right)C_{i}$$

(5) $$\frac{\text{dAdd}}{dt}=k_{\mathrm{add}}C_{i}$$

where $V_{o}$, $V_{i}$ and ${\text{pH}}_{e}$, ${\text{pH}}_{i}$ are respectively the volumes and pH values of the extra‐ and intracellular compartments, $p_{T}$ and $p_{T2}$ are TMZ uptake and efflux rate constants, respectively, $k_{T}\left(\text{pH}\right)$ and $k_{M}\left(\text{pH}\right)$ are the pH‐dependent rate constants of TMZ transformation into MTIC and subsequent MTIC activation into the cation C, $k_{\mathrm{cat}}$ is the cation degradation rate constant which presents a high reactivity, and $k_{\mathrm{add}}$ is the DNA‐adduct formation rate constant. As in Ballesta et al.,^4^ $k_{T}\left(\text{pH}\right)$ and $k_{M}\left(\text{pH}\right)$ are modeled as follows:

(6) $$k_{T}\left(\text{pH}\right)=k_{T}^{0}e^{\lambda_{T}\cdot \text{pH}}$$

(7) $$k_{M}\left(\text{pH}\right)=k_{M}^{0}e^{-\lambda_{M}\cdot \text{pH}}$$

where ($k_{T}^{0}$, $\lambda_{T}$) and ($k_{M}^{0}$, $\lambda_{M}$) are non‐physiological parameters to estimate. All model parameters were estimated from in vitro studies in buffer solutions or in U87 glioma cells and the best‐fit model achieved a very good fit to data^4^; Table 1).

**Table 1:** Model parameters of TMZ PK, all taken from the original ‐experimentally validated‐ model by (4), except parameters with symbol (*) that were rescaled as described in Appendix A

Parameters	Unit	Value	Description
${\text{TMZ}}_{0}$	μmol/L	62	Initial TMZ concentration
${V_{i}}^{*}$	l	8e^−12^	tumor cell volume
${V_{o}}^{*}$	l	25e^−6^	Extracellular volume
${p_{T}}^{*}$	l/h	4.34e^−9^	TMZ influx
${p_{T2}}^{*}$	l/h	9.14e^−9^	TMZ efflux
$k_{T}^{0}$	$h^{-1}$	1.1e^−7^	TMZ metabolism into MTIC
$\lambda_{T}$	$h^{-1}$	2.09	
$k_{M}^{0}$	$h^{-1}$	292	MTIC metabolism into cations
$\lambda_{M}$	$h^{-1}$	0.31	
$k_{\mathrm{cat}}$	$h^{-1}$	6000	Methylating cation degradation
$k_{\mathrm{add}}$	$h^{-1}$	0.005	DNA adducts formation rate

### 2.2 Spatial hybrid model of TMZ PK‐PD

In order to account for tumor heterogeneities depending on spatial cell location in its environment or inter‐cell variability, the ODE‐based model was coupled to a spatial cellular automaton (CA). The CA gives an explicit representation of each individual cell in the tumor and this allows to relate each cell to its environment in terms of other cells and of extracellular molecular species (ie, oxygen, hydrogen ions, chemotherapeutic drugs, etc.). The CA is defined as a two‐dimensional grid with 200 × 200 square elements representing a 25$mm^{2}$ area. Each tumor cell can occupy one element $\left(i,j\right)$ of the grid with dimensions Δx=Δy. The binary variable c is set as $c_{i,j}=1$, if there is one tumor cell at location (i,j) and 0 otherwise. The tumor cell population in the CA is thus defined as follows:

(8) $$C=\{\left(x_{i},y_{j}\right)|c_{i,j}=1,x_{i}=i\Delta x,y_{j}=j\Delta y\}$$

TMZ intracellular PK‐PD is solved for each tumor cell using the ODE‐based model (equations (2), (3), (4), (5)). No inter‐variability is considered in the model parameters. The extracellular TMZ dynamics is now represented by a Partial Differential Equation (PDE) assuming an uniform spatial diffusion of the drug in the extracellular environment (equation (9)). Reaction terms are added to represent TMZ cellular uptake and efflux, together with its pH‐dependent transformation into MTIC in the extracellular medium. Extracellular spatiotemporal TMZ dynamics, $To\left(x,y,t\right)$, is thus given by the following PDE:

(9) $$\frac{\partial T_{o}}{\partial t}=D\nabla^{2}T_{o}-k_{T}\left(\mathrm{pH}\right)T_{o}\underset{\;\text{if}\;\left(x,y\right)\in C}{\underbrace{-\frac{p_{T}}{V_{o}}T_{o}+\frac{p_{T2}}{V_{o}}T_{i}}}$$

where $D=1.7\times 10^{-5}$ cm^2^/s is the TMZ diffusion coefficient^20^ and $V_{o}$ is the volume of the extracellular medium (Appendix A). TMZ transport into/from the cells only occurs at spatial location occupied by cells. The intracellular concentrations of TMZ ($T_{i}$), MTIC ($M_{i}$), cations ($C_{i}$) and DNA‐adducts (Add) are now cell‐dependent (ie, space‐dependent).

As a preliminary test, we reproduced with the hybrid model the PK results obtained with the non‐spatial model which were based on experimental measurements in U87 cultured cells^4^ (Appendix B). For that, we considered a circular tumor mass of 8000 cells (approximating 20% confluence of the CA grid). As simulations were performed on a duration of less than 10 hours, we disregarded cells proliferation and cell death on this short time scale. This benchmarking test validated the scaling stage of the hybrid model parameters (Appendix A).

### 2.3 Modeling pH evolution

TMZ PK being largely driven by local pH values,^4^, ^6^ it is important to adequately represent the extra‐ and intracellular pH for both tumor and healthy cells. In all in vitro studies, the extracellular pH was assumed to be constant and uniform across the Petri dish since the cell culture medium is theoretically a buffer solution aiming to maintain constant pH values. At the opposite, in the in vivo context, the pH depends on the cells presence and on the vascular environment. This results in the appearance of large spatial heterogeneities.

#### 2.3.1 Extracellular pH

One characteristics of the tumor cells is their high level of glucose consumption.^21^ Positron emission tomography (PET scan) is now standardly used to highlight the tumor sites thanks to this specific signature.^22^, ^23^ The increased glycolytic metabolism is related to hypoxia: as the tumor grow the increased cell population increases oxygen consumption and contribute to the local vascular disruption that together result in a local deficit of oxygen. To survive, the cells switch their metabolism to glycolysis. Even if oxygen levels come back to normal, tumor cells tend to favor the glycolytic pathway as the source of energy, this is known as the Warburg effect.^24^ Hydrogen ions are by‐products of glycolysis and accumulate in and around the tumor since they are not efficiently washed out by the locally damaged vascular network. This contributes to build up the acidic tumor environment. Some models explicitly describe the hydrogen ions production by the tumor cells,^7^, ^15^ however to make things simple we made the choice to directly index the level of acidity to the level of hypoxia since both phenomena are very often co‐localized in tumors. On the long term, local re‐oxygenation of the tissue, through angiogenesis, might not necessary lead to a decreased acidity in the same proportion because of the Warburg Effect,^25^ however since we will only consider short‐term events (of a few hours) in this study, we assume that the oxygen level can be taken as a good indicator of local acidity. Therefore, we constructed a function that directly gives the extracellular pH given the stationary oxygen concentration since this quantity can easily be computed through the cellular automaton^26^, ^27^ (Appendix C).

Given the local oxygen concentration (Oxy) and a threshold value for oxygen ($\text{Ox}y_{\mathrm{thr}}$) below which the pH is assumed to saturate to its minimum pH${\;}_{\min}$ (due to the limited production rate of H+ by the cells), the pH is computed as follows:

(10) $$\;\text{if}\;\text{Oxy}>\text{Ox}y_{\mathrm{thr}}\;\text{then}\;\;\text{pH}\;=\;\text{pH}{\;}_{\max}-\frac{\Delta \;\text{pH}\;}{\Delta \text{Oxy}}\left(\text{Ox}y_{\max}-\text{Oxy}\right)$$

(11) $$\;\text{else}\;\;\text{pH}\;=\;\text{pH}{\;}_{\min}$$

where pH${\;}_{\max}$ is the pH in normal healthy tissues (ie, normally oxygenated tissue, corresponding to $\text{Ox}y_{\max}$) and is typically 7.4 and pH${\;}_{\text{min}}$ is the lower pH level found in tumors which can be as low as 6.5.^28^, ^29^ We set these two values to pH${\;}_{\max}$ and pH${\;}_{\min}$ respectively. $\Delta \text{Oxy}=\text{Ox}y_{\max}-\text{Ox}y_{\mathrm{thr}}$ and $\Delta \;\text{pH}\;=\;\text{pH}{\;}_{\max}-\;\text{pH}{\;}_{\min}$ (Appendix Figure A2).

#### 2.3.2 Intracellular pH

One hallmark of the tumor cells is their ability to survive in an acidic environment – that they contribute to generate – by maintaining their intracellular pH at physiological levels. On the other hand, this acidic environment is detrimental to normal cells that have not acquire this ability.^28^ Intracellular pH regulation is a complex process that is not completely elucidated yet.^30^, ^31^ However, simultaneous measurements of extra and intracellular pH were made in several tumor cell types that all exhibit the reversed pH property where the intracellular pH is higher than the extracellular one.^30^, ^32^, ^33^, ^34^

**Figure 3:**
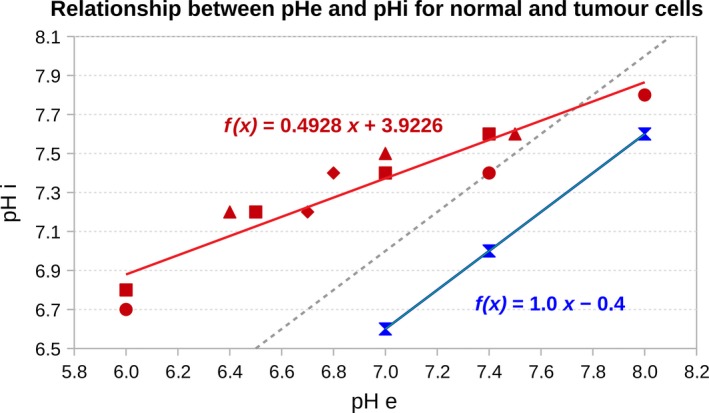
Relationship between ${\text{pH}}_{e}$ and ${\text{pH}}_{i}$ for normal and tumor cells. The g(x) function corresponds to normal cells and is derived from the physiological status point (sandglass point). We consider that ${\text{pH}}_{i}$=${\text{pH}}_{e}-0.4$ as indicated by the function.^39^ Since normal cells are not able to survive acidity, the function g(x) is only valid from ${\text{pH}}_{e}=7$ under this value we consider that the intracellular acidity is lethal to the cell. The f(x) function is a linear regression estimated from the points corresponding to different tumor cell types: SCK cells (bullets),^34^ CC139 cells (squares),^33^ PANC‐1 cells (triangles),^32^ other tumor cells (diamonds). The dotted line indicates where ${\text{pH}}_{e}$ = ${\text{pH}}_{i}$

For this study, we needed to evaluate the intracellular pH given the extracellular one. To that end, we compiled from the literature intra and extracellular measurements performed on different cell types that were available for a wide range of extracellular pH. The different points obtained from four different studies, corresponding to four different tumor cell types: mice mammary carcinoma (SCK),^34^ Chinese hamster lung fibroblasts (CC139),^33^ human pancreatic carcinoma (PANC‐1),^32^ general tumor cells^30^ could be fitted by linear regression to calculate the coefficients $\left(a,b\right)=\left(0.4928,3.9226\right)$ to give the pH${\;}_{e}$‐pH${\;}_{i}$ relationship for tumor cells (Figure 3, $f\left(x\right)$):

(12) $$\;\text{pH}{\;}_{i}=a\times \;\text{pH}{\;}_{e}+b$$

For normal cells, the physiological status point was well characterized in different cell types with pH${\;}_{e}$=7.4 and pH${\;}_{i}$=7.^35^, ^36^, ^37^, ^38^ The intracellular pH is found to evolve passively with the extracellular pH, being around half a unit lower^39^ which gives $\left(a,b\right)=\left(1,0.4\right)$ for normal cells (Figure 3, $g\left(x\right)$).

### 2.4 Data and Statistical analysis

The simulations for the in vitro settings were realized with Matlab (version 9.1, R2016b). The hybrid model was developed as a graphical Microsoft application under Visual Studio.net 2003 using a C++ compiler. The ODE was solved with an Euler numerical scheme. The PDE was solved using the Thomas Algorithm (for tridiagonal matrices) and the Alternate Direction Implicit (ADI) Method to solve the diffusion equation in each of the two space dimensions alternatively. The same numerical grid was used to solve the PDE and to implement the cellular automaton. Data and statistical analysis comply with the recommendations on experimental design and analysis in pharmacology.^40^

## 3 RESULTS

### 3.1 TMZ pH‐dependent PK‐PD in tumor and normal cultured cells

**Figure 4:**
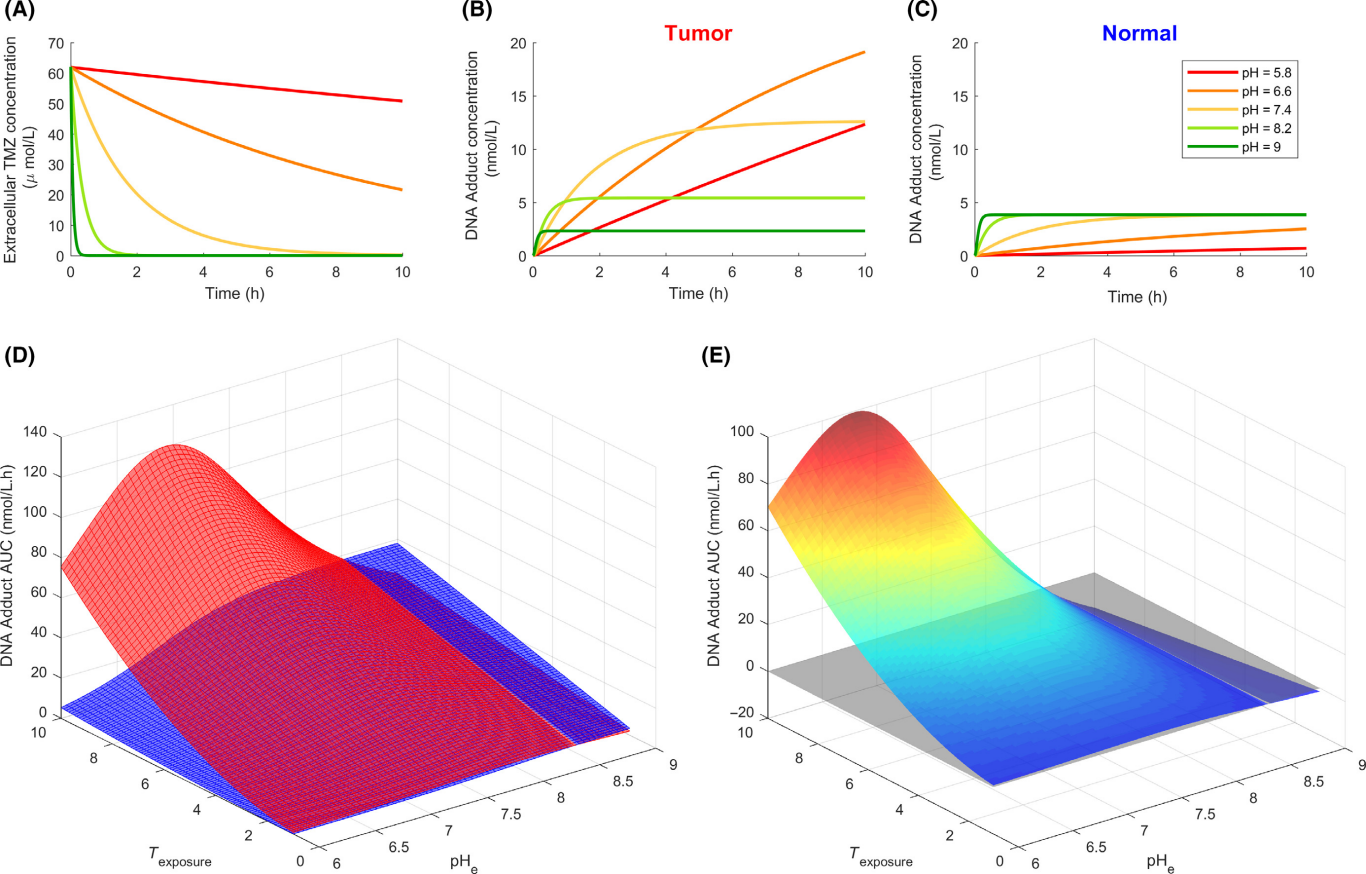
pH‐dependent TMZ PK‐PD in tumor and normal cultured cells. (A) Extracellular TMZ concentration time profiles for various extracellular pH values; (B) Intracellular concentration of DNA adducts in tumor cells for various extracellular pH values; (C) Intracellular concentration of DNA adducts in normal cells for various extracellular pH values; (D) DNA adduct AUC values for various extracellular pH values and TMZ exposure duration in tumor (upper curve) or normal (lower curve) cells; (E) Difference in DNA adduct AUC values between tumor and normal cells

We first used TMZ PK‐PD ODE‐based model to investigate the drug pharmacology in the context of an in vitro setting. In this scenario, two cell types corresponding to normal and tumor cells were assumed to be cultured as monolayers ensuring a uniform pH and access to the drug. TMZ exposure concentration was set to 60 μmol/L. Extracellular pH values were assumed to be constant as culture media are theoretically buffer solutions. Under acidic conditions, TMZ was hardly transformed into MTIC (Figure 4A). As pH increased, its stability decreased to become extremely unstable with a complete degradation in less than 2 hours for pH above physiological values. Although normal and tumor cells were exposed to the same extracellular TMZ concentrations, TMZ pH‐dependent PD appeared radically different in the simulations for normal and tumor cells as a result of different intracellular pH regulation (Figure 4B,C, see Materials and Methods). In normal cells, intracellular pH (pH${\;}_{i}$) followed pH${\;}_{e}$ with an acidic shift. As a result, the amount of DNA‐adducts in the normal cells remained to very low levels for acidic pH${\;}_{e}$ due to TMZ neutralization. In the same acidic conditions, the amount of DNA adducts in the tumor cells built up much higher as a result of higher pH values in the intracellular compartment as compared to healthy cells. Interestingly, at physiological pH, that is*,* pH${\;}_{e}$=7.4, DNA adduct concentration was 3.5‐fold higher in tumor than in normal cells. When pH${\;}_{e}$ was maintained above physiological levels, DNA damage in the tumor cells were in the same range as that in normal cells. To further investigate optimal pH values, we computed the cumulative amount of DNA damage over the entire TMZ exposure in the form of the Area under the curve (AUC) of DNA adduct concentration. For any pH${\;}_{e}$ values between 6 and 8.5, TMZ‐induced DNA damage was larger in tumor cells as compared to healthy cells (Figure 4D). For all considered exposure durations, maximum antitumor efficacy was computationally obtained for pH values between 6.65 and 7.9, optimal pH decreasing with exposure time. Optimal pH values inducing the largest difference between DNA damage in tumor and healthy cells were comprised between 6.8 and 7.5 and decreased with exposure duration (Figure 4E).

### 3.2 TMZ pH‐ and space‐dependent PK‐PD in an in vivo solid tumor

The next step was to computationally investigate TMZ PK‐PD in a solid tumor within its environment. Tumor tissues may present (a) modified micro‐environment and in particular abnormal vascular network, (b) different spatial configurations. In this section, we intend to specifically study these two elements and their influence on local pH and TMZ PK‐PD. Our hybrid approach that involves a cellular automaton is particularly well adapted to that task since the model simultaneously accounts for spatialization and cell individualization.

#### 3.2.1 TMZ PK‐PD dependency on tumor microenvironment

**Figure 5:**
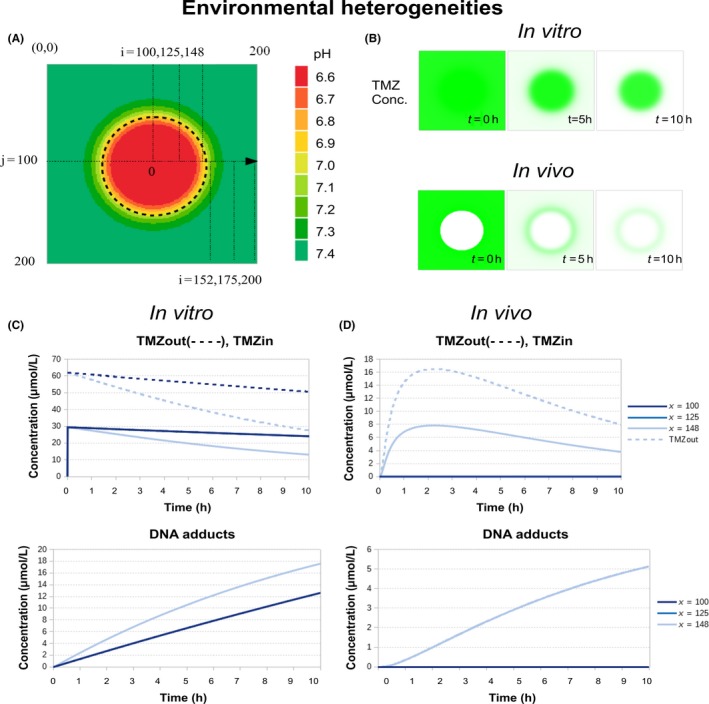
The two considered sources of heterogeneity in the medium. (A) pH spatial variations (the dotted line represents the tumor boundary); (B) temporal evolution of TMZ concentration for a homogeneous capillary network and for a degraded one. In the homogeneous case, TMZ is initially (t = 0 h) homogeneously distributed whereas when the capillary network is degraded inside the tumor mass, the tumor does not have access to TMZ initially. Comparison of TMZ PK depending on the cell location in the tumor spheroid, for the homogeneous capillary network (left column) and for the degraded one (right column). The PK is represented for three cells located at three different distance x from the center of the 2D tumor: x=0 (centre), x=25 (intermediate), x=48 (periphery) (tumor radius R=50 grid units) as shown in (A)

We here considered two major sources of environmental heterogeneities that can impact on TMZ PK‐PD: the pH that affects the successive stages of TMZ transformation into its active compound, and the tumor vascularization that affects TMZ delivery in the tissues. The resources (oxygen, nutrients) and the drugs are delivered through the capillary network. In a normal healthy tissue, this network is homogeneously covering the volume of the tissue. On the opposite, in tumors, the capillary network is degraded: (i) vessels are crushed by the proliferating tumor cells, (ii) the increased acidity triggers apoptosis of the normal endothelial cells, (iii) growth factors destabilize the capillaries by stimulating vessels sprouting for angiogenesis.^27^ As a consequence, two scenari were simulated with the model: (a) the capillary network was considered to be intact and TMZ was initially homogeneously distributed to the tumor cells, (b) the capillary network was degraded, TMZ was delivered from the intact capillary network outside of the tumor mass which then diffused to reach the tumor cells (Figure 5B, *t* = 0 h). In reality, the capillary network may be mostly degraded inside the tumor, although not completely destroyed. We compared these two extreme cases to better highlight the consequences on TMZ PK‐PD.

Simulations were performed for a circular tumor mass of radius R=50 units (corresponding to 8000 cells), centerd in the cellular automaton grid $\left(i,j\right)=\left(100,100\right)$. The pH spatial variations associated to the avascularized tumor mass was first computed which revealed a gradient from the center of the tumor to its periphery as a result of local oxygen concentration (Figure 5A, Appendix C). pH distribution for the vascularized tumor was assumed to be the same as the avascularized tumor so as to uncouple the effects of acidity and TMZ access. In terms of biology, this illustrates the Warburg effect by which tumor cells favor glycolysis which is the source for a sustained acidity even in the presence of oxygen delivered through a functional capillary network. For the intact vasculature, TMZ was rapidly transformed into MTIC at the tumor periphery which is at physiological pH $\left(x_{i}=148\right)$ but was stabilized at the center of the tumor $\left(x_{i}=100\right)$, because of the local acidity (Figure 5B, Homogeneous capillary network). In the case of a degraded vasculature, TMZ diffusion was too slow to allow for drug penetration inside the tumor before its transformation into MTIC in peripheral regions at physiological pH. Interestingly, TMZ was stabilized by the acidity at the tumor periphery as shown by the TMZ ring surrounding the tumor that slowly faded away because of diffusion (Figure 5B, Degraded capillary network).

TMZ PK‐PD results were presented for three cells located at three different points in the tumor from the center to the periphery: $\left(x_{i},y_{j}\right)=\left(100,100\right)$, $\left(x_{i},y_{j}\right)=\left(125,100\right)$, $\left(x_{i},y_{j}\right)=\left(148,100\right)$ and were compared between the intact vasculature and the degraded one (Figure 5C,D). In both the vascularized and avascularized tumors, no differences in TMZ PK‐PD were observed for the two most inner locations ($x_{i}$=100 and 125) and curves were superimposed, as a result of environmental conditions in terms of pH and TMZ exposure being close, the peripheral case ($x_{i}$=148) was different though. For the homogeneous capillary network, the drug was more efficient at the periphery where the pH is closer to the physiological level. DNA adducts built up more rapidly for these peripheral tumor cells as compared to cells located inside the tumor (Figure 5C). For the degraded capillary network, TMZ PK was altered as the drug that diffused from outside of the tumor only reached the peripheral cells and most of tumor cells were unaffected due to TMZ slow diffusion (Figure 5D). The kinetics was slower than that of the vascularized tumor with 3.5‐fold less DNA adducts produced after 10 hours of drug exposure.

These simulation results showed that both pH and the state of the capillary network are essential determinants of TMZ PK‐PD. Interestingly, the concomitant effect of the tumor pH gradient and of the poor quality of the tumor vascular network participated in reducing TMZ efficacy.

#### 3.2.2 TMZ PK‐PD in different tumor spatial configurations

**Figure 6:**
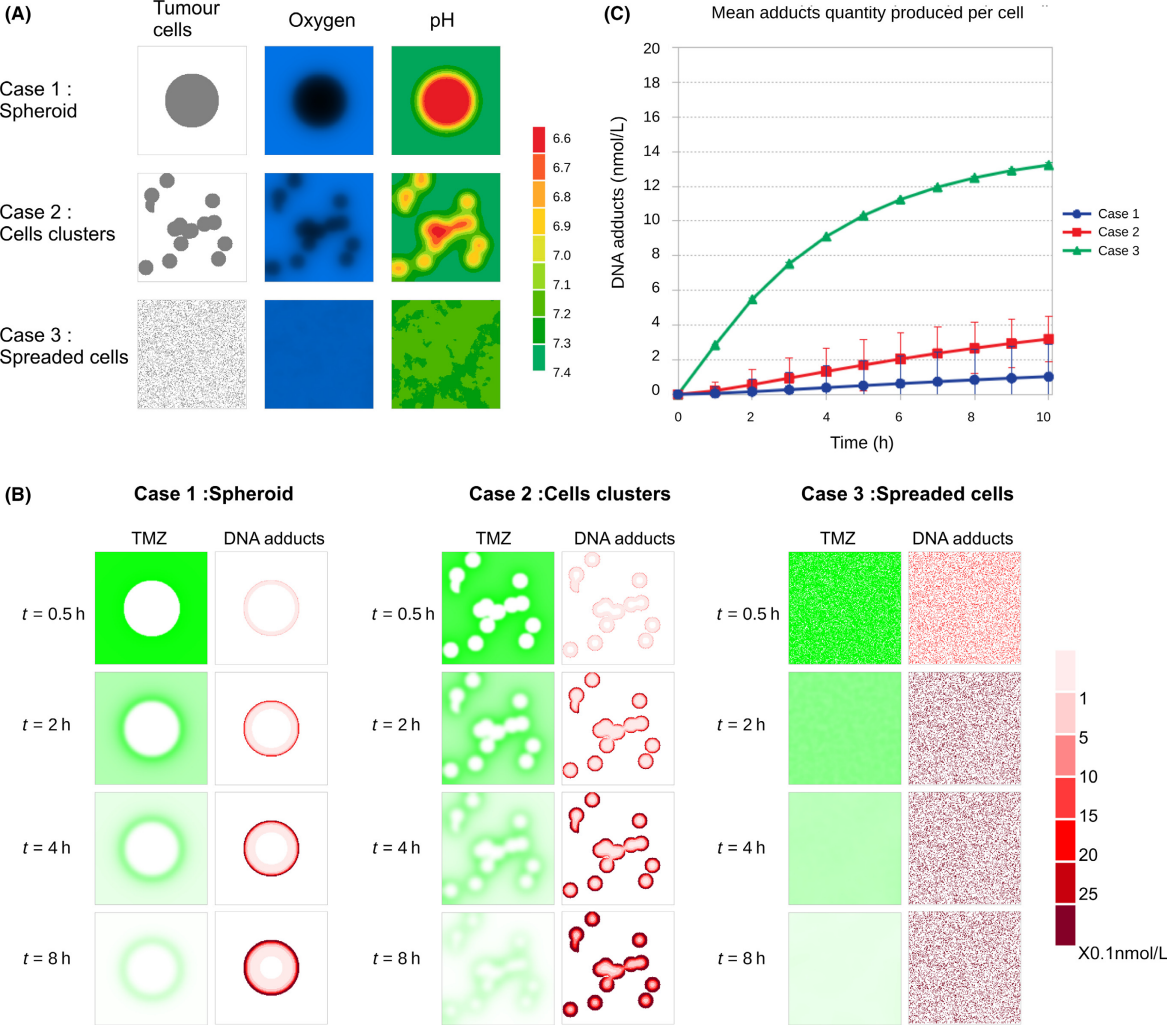
Comparison of different tumor conformations. (A) tumor cells in the three different conformation cases: (1) spheroid, (2) cells clusters, (3) spread cells; corresponding oxygen concentration map; corresponding extracellular pH. (B) TMZ degradation/uptake (first columns) and associated DNA‐adducts accumulation in tumor cells (second columns) for the three tumor conformations. (C) mean amount of DNA adducts accumulation per cell for the three different tumor configurations. Note: the mean amount of DNA adducts is calculated over the all tumor cell population for each case

Different tumor spatial configurations may be observed depending on the tumor stage, cancer cell migration phenotypes, extracellular matrix properties, etc. Those may be associated to an increased tumor aggressiveness where the tumor tends to split into clusters and/or individual tumor cells escape from the tumor mass to invade surrounding tissues. This is a typical feature of glioblastoma where tumor cells often disseminate in the healthy brain tissue.^41^ Therefore, we here considered three different tumor configurations to better assess the importance of spatiality: a spheroid (case 1, as before), cell clusters (case 2), spread cells (case 3) (Figure 6A). In the three cases, the total amount of cells was conserved. Furthermore, we considered a degraded capillary network so that TMZ was initially only in the tumor peripheral tissues. As before, pH distribution was first computed for each tumor configuration from the oxygen local concentration (Figure 6A). Simulations were made ‐ using the same set of parameters for all three cases—to evaluate the in vivo availability of TMZ and its impact based on DNA adduct generation (Figure 6B). For all three cases, tumor cells were not all homogeneously affected, even after 10 hours of drug exposure. Inside the tumor mass could even totally escape from treatment in the case of a spheroid or cell clusters. The spheroid case was the less favorable in that sense, as it led to the smallest averaged DNA adduct intracellular concentrations, which was evaluated over the whole tumor cell population (Figure 6C, bullets). Next, inter‐cell Standard Deviations (SD) of DNA adduct concentrations were large as a result of spatial heterogeneity in the spheroid scenario. Cell aggregates also harbored unaffected cells with both a slightly increased mean DNA adduct level and slightly decreased heterogeneity between cells as compared with the spheroid case (Figure 6C, squares). Finally, spread cells were all homogeneously targeted with mean DNA adduct over the whole cell population being approximately 14‐fold higher than in the spheroid case after 10 hours of TMZ exposure (Figure 6C, triangles).

### 3.3 Influence on TMZ PK‐PD of inter‐cell variability in drug cellular transport

One important hallmark of cancer cells is their large inter‐cell heterogeneity regarding intracellular gene and protein levels. We used our models of TMZ PK‐PD to assess the impact of inter‐cell variability on the drug response. In TMZ PK‐PD, TMZ transformation into MTIC and MTIC conversion into the active cation are spontaneous reactions which are unlikely to present large inter‐cell variability. On the opposite, TMZ cellular transport may be mediated by active transporters which could display different expression levels across the tumor cell population. Hence, we studied TMZ PK‐PD in a heterogeneous cell population presenting variability in TMZ cellular uptake and efflux.

#### 3.3.1 Inter‐cell variability in an in vitro setting in acidic conditions

**Figure 7:**
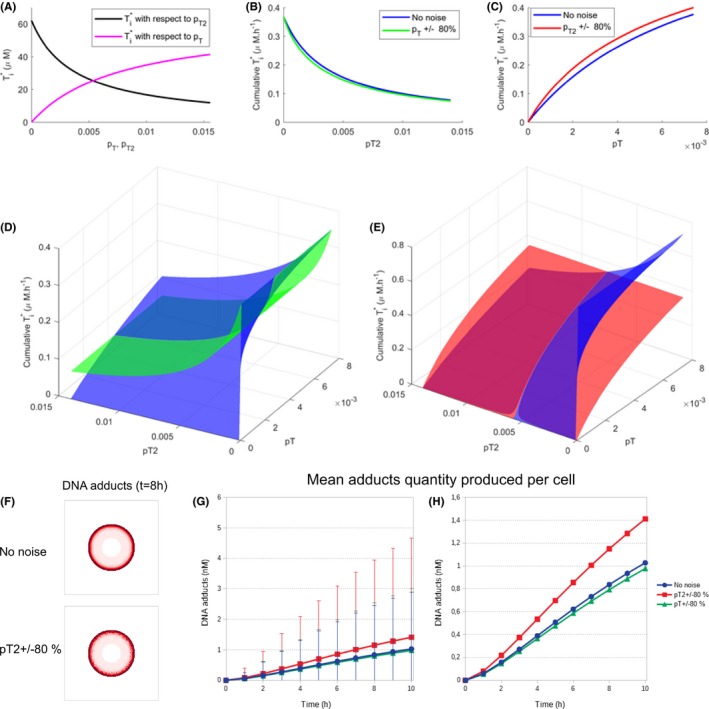
Effect of TMZ transport parameters variability in cells. (A) TMZ intracellular concentration at steady state with respect to TMZ uptake parameter ($p_{T}$) or efflux parameter ($p_{T2}$); (B) TMZ cumulative steady state intracellular concentration in a homogeneous cell population (no noise) or a heterogeneous population presenting inter‐cell variability in TMZ uptake ($p_{T}$ ± 80%); (C) TMZ cumulative steady state intracellular concentration in a homogeneous cell population (no noise) or a heterogeneous population presenting inter‐cell variability in TMZ efflux ($p_{T2}$ ± 80%); (D) TMZ cumulative steady state intracellular concentration with respect to $p_{T}$ and $p_{T2}$ in a homogeneous cell population (dark curve) or a heterogeneous population presenting inter‐cell variability in TMZ uptake (light curve); (E) TMZ cumulative steady‐state intracellular concentration with respect to $p_{T}$ and $p_{T2}$ in a homogeneous cell population (dark curve) or a heterogeneous population presenting inter‐cell variability in TMZ efflux (light curve);(F) DNA adducts accumulation in the perturbed and non‐perturbed cases; (G) mean amount of DNA adducts accumulation per cells for perturbed inflow ($p_{T}$) and outflow ($p_{T2}$) parameters; (H) close up of (G) with standard deviation (SD) bars removed. Note: the mean amount of DNA adducts is calculated over the all tumor cell population for each case

For this simulations, we assumed an in vitro setting in which cells have uniformly access to TMZ and in which pH is constant and equal to an acidic value. In acidic conditions, TMZ is not metabolized and the sole reactions occurring were the drug cellular uptake and efflux. Since TMZ is stabilized, we assumed that TMZ total quantity was conserved and equal to $T_{\mathrm{tot}}$ so that $T_{i}\left(t\right)+T_{o}\left(t\right)=T_{\mathrm{tot}}$. The steady state of TMZ intracellular concentration $T_{i}^{*}$ can then easily be derived from equations ((1) and (2)), and is equal to:

(13) $$T_{i}^{*}\left(p_{T},p_{T2}\right)=\frac{p_{T}T_{\mathrm{tot}}}{p_{T}+p_{T2}}$$

where $p_{T}$ and $p_{T2}$ are the rate constants of TMZ cellular uptake and efflux, respectively. Interestingly, TMZ intracellular steady state level varied with respect to $p_{T}$ and $p_{T2}$ in a non‐linear manner (Figure 7A). We then studied a heterogeneous cell population in which each cell displayed a different value of $p_{T}$. The parameter values were uniformly selected in the interval $\left[0.2p_{T}^{†},1.8p_{T}^{†}\right]$ corresponding to a deviation of 80% of the value $p_{T}^{†}$ estimated from experimental data in U87 cells. TMZ cumulative intracellular steady state in the whole cell population was computed by integrating $T_{i}^{*}$ with respect to $p_{T}$ on the studied interval and the same quantity was computed for a homogeneous cell population with no inter‐cell variability (Appendix D). TMZ intracellular steady state level was predicted to be similar in the homogeneous cell population and in the cell population presenting variability in TMZ uptake when $p_{T2}$ was set to its value estimated from U87 data $p_{T2}^{†}$ and $p_{T}$ was varied around its data‐derived value (Figure 7B). However, simulations for different values of the fixed parameter $p_{T2}^{†}$ and of mean $p_{T}$ yielded different results (Figure 7D). For small $p_{T}$ mean values, TMZ accumulated more in the heterogeneous cell population compared to the homogeneous one whereas for $p_{T}$ mean values larger than some threshold value, the situation was reversed. $p_{T}$ threshold value increased with $p_{T2}^{†}$ and was comprised between 0.0025 and 0.0035 $h^{-1}$. The same study was performed for the parameter $p_{T2}$ which was varied uniformly in $\left[0.2p_{T2}^{†},1.8p_{T2}^{†}\right]$, $p_{T2}^{†}$ denoting the value estimated from data. Interestingly, variability in the parameter $p_{T2}$ increased the cumulative intracellular concentration of TMZ present in the cell population at steady state (Figure 7C). Again, this result was dependent on the chosen values for the fixed parameter $p_{T}$ and the varied one $p_{T2}$ (Figure 7E). For small $p_{T2}$ mean values, TMZ accumulation was larger in the homogeneous cell population as compared to the population with inter‐cell variability in TMZ efflux. When $p_{T2}$ was larger than a threshold value in [0.0056, 0.0073], increasing with $p_{T}^{†}$ value, the situation was reversed.

#### 3.3.2 Inter‐cell variability in a solid tumor

We then used the hybrid model to investigate the impact of inter‐cell variability on TMZ PK‐PD in a solid tumor. As for the in vitro study, we varied $p_{T}$ and $p_{T2}$ parameters which correspond to TMZ uptake and efflux rate constants, respectively. One advantage of using a cellular automaton was the possibility to test variability in individual cell properties. Thus, we randomly assigned to each cell a perturbed value of $p_{T}$ and $p_{T2}$ separately (ie*,* in two different simulations). Different perturbation amplitudes were considered and the effects only started to be visible for ±80% amplitude (Figure 7F,G,H). Interestingly, the impact of these two parameters was very close to the results in the in vitro setting. Perturbation of the efflux parameter $p_{T2}$ produced a bigger impact on the resulting mean DNA adducts level in the whole cell population. On the other hand, the impact of varying $p_{T}$ was small compared to the unperturbed case with only a slight decrease in the mean DNA adducts concentration.

## 4 DISCUSSION

Although pH is known to critically influence TMZ PK‐PD,^4^ its potential impact on the drug efficacy has not yet been fully investigated. Thus, we designed a complete theoretical framework to study TMZ PK‐PD in both in vitro and in vivo settings, incorporating pH dependency, spatial heterogeneities, and inter‐cell variability in TMZ transport. Overall, all simulations in all scenari predicted that optimal TMZ efficacy was obtained when tumor pH was close to physiological pH. This is clearly an argument for considering pH as a therapeutic target and advocates for future research on the combination of TMZ with pH‐regulating agents.

We first evaluated the differential response to TMZ of tumor and healthy cells presenting different intracellular pH regulations. Interestingly, the model provided quantitative predictions regarding the drug differential impact on normal or cancer cells and optimal pH values leading to an advantage for healthy cells. Tumor cells were able to maintain relatively high intracellular pH in acidic environment, whereas normal cells were assumed to regulate their pH proportionally to the extracellular one. As a consequence, at the same extracellular pH, both cell types presented different sensitivity to TMZ. Indeed, our simulation results showed that, for extracellular pH between 5.8 and 8.2, TMZ transformation into its active compound and subsequent DNA damage were larger in tumor cells as compared to normal cells thanks to less acidic intracellular pH in cancer cells. This indicated that the local acidity often encountered in tumor tissues was still a favorable ground for TMZ effectiveness. However, optimality of TMZ efficacy defined as a maximum difference between drug‐induced damage in tumor and healthy cells was reached between 6.8 and 7.5 which is closer to physiological values.

Next, we computationally investigated TMZ PK‐PD in a solid tumor taking into account its environment. In vivo cells are not homogeneously exposed to the same pH, nor to the same drug concentration. These essentially depend on the location of the cells in the tumor tissue (central parts *versus* periphery). Non‐spatial PK‐PD approaches only describe temporal aspects assuming that the whole cell population is homogeneously exposed to the same environmental conditions and reacts in the same way to the drug. To overcome these limitations, we proposed a hybrid framework incorporating a cellular automaton that allowed to explicitly compute TMZ PK‐PD for each individual tumor cell given its particular local environment. Our simulations showed that spread cancer cells or fragmented tumors presented higher TMZ‐induced damage as compared to compact tumor spheroid. The model provided insights into molecular explanations for this result. First, pH values close to normal in smaller tumors micro‐environment allowed for TMZ activation whereas bigger tumors were more acidic and prevented the drug from transformation into its active metabolites. This potential mechanism of resistance to TMZ has not been described in the literature up to our knowledge. The model also confirmed as expected that isolated cancer cells or fragmented tumors could be reached by the drug whereas, at the heart of a tumor spheroid, the most inner cells had no access to the drug due to damaged vascular network and insufficient drug diffusion in the interstitial fluid.

Our in vivo simulations were performed for a generic solid tumor as TMZ is approved for the treatments of several types of malignancies, although the drug is mainly used against brain tumors. To specifically represent a brain tumor and surrounding healthy brain tissues, one needs to incorporate the blood–brain barrier (BBB) along the capillaries. While the BBB is intact in the normal tissues and decreases TMZ penetration rate in the interstitial fluid, the barrier is often altered at the neighborhood of the tumor mass where the combination of acidity, growth factors stimulating angiogenesis and increased cell density destabilize blood vessels and increase their permeability. Adding the BBB component to our hybrid model may modify TMZ efficacy on spread cells if they are located in normal tissues where the BBB is too weakly altered to allow for TMZ brain penetration.

In the last paragraph, we considered the importance of inter‐cell variability in TMZ cellular transport. We provided a counter‐intuitive prediction regarding a differential effect of inter‐cell variations on TMZ uptake or efflux, respectively. Indeed, for model parameters corresponding to U87 glioma cells, inter‐cell variations of the uptake parameter play no role regarding the mean amount of DNA adducts in the whole cell population. On the opposite, variations of the efflux parameter let to a significant increase of the DNA damages induced by the TMZ. Indeed, cells with a reduced efflux dramatically increased their level of DNA adducts which overcame the impact of the increased efflux in other cells. Hence, inter‐cell variability in TMZ efflux was a disadvantage for the tumor cell population in terms of drug resistance. Exploring other parameter values revealed that this conclusion may not hold for other cell types presenting different kinetics for TMZ uptake and efflux.

As a conclusion, our study showed that employing new modeling approaches enabled us to tackle the full complexity of a drug pharmacology and thus inform on drug optimal scheduling and combinations. This is further advocated by the fast developing fields of systems oncology and systems pharmacology.^41^, ^42^ Specifically, our model has allowed to show that pH and drug access are determining factors and depend on the cell type and tumor configuration which gives fundamental insights to decipher the impacts of interrelated conditions. On the prospective plan, the model highlights potential means to enhance the TMZ efficacy by acting dynamically on the local pH.^10^ The realization of which opens up new challenging paths for research with potential high benefit for patients.

## AUTHOR CONTRIBUTIONS

A.S. and A.B performed the simulations that concern the in vivo and in vitro cases, respectively. Both authors equally contributed in the writing of the manuscript.

## DISCLOSURE

None.

## Figures and Tables

**Figure B1:**
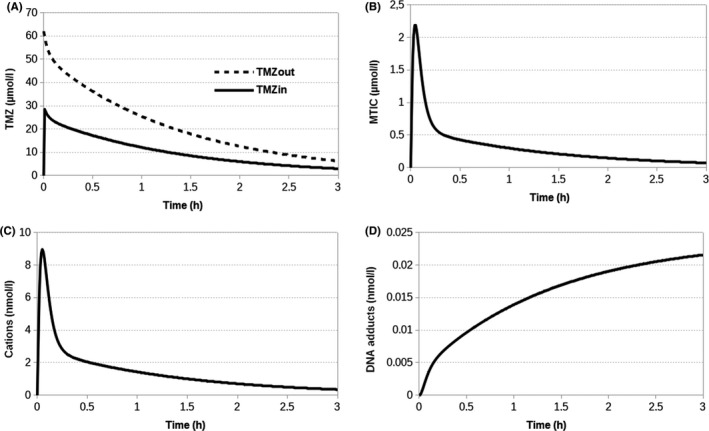
Agreement of the hybrid model with the initial ODE‐based model. (A) TMZ and (B,C,D) intracellular MTIC, Cation and DNA adducts PK, respectively, for any tumor cell of the cellular automaton. These kinetics reproduce the results presented in Ballesta et al.,^4^

**Figure C1:**
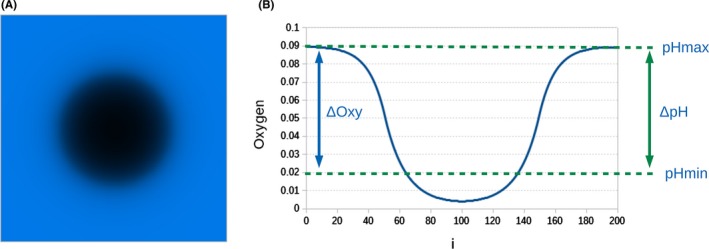
Relationship between oxygen and pH. (A) The stationary state of oxygen concentration is first calculated for a spheroid of a given radius (R=50 units). The tumor boundary is materialized by the dotted circle line. (B) The stationary oxygen profile along a line passing through the middle of the spheroid is given by the U‐shape curve. The minimum for the oxygen concentration (in arbitrary units) is reached at the center of the tumor (which corresponds to i=100 units). Since H+ production by the cells is limited it is here assumed to saturate beyond a threshold level of oxygen ($\text{Ox}y_{\mathrm{thr}}$) indicated by the lower dashed line. The upper level of oxygen ($\text{Ox}y_{\max}$) correspond to the normal physiological level of oxygen in the tissue. The oxygen variation between these 2 levels (ΔOxy) is assumed to correspond to the maximum pH variation between the center of the tumor and the surrounding tissue (ΔpH)

## APPENDIX A

### PARAMETERS RESCALING

#### Volumes

In the original ODE model,^4^ the extracellular medium is covering in vitro an overall population of $10^{6}$ tumor cells. The volume of the medium, that is, the extracellular volume, is taken as $V_{o}=2\text{mL}$. The volume of each single cell is approximately 7*pL* so the total intracellular volume is $V_{i}=7\mu L$.

In the cellular automaton, the simulation domain is taken as a 5 mm long square box. The extracellular medium is assumed to cover the cells in the automaton grid up to 1mm high. The extracellular medium thus represents a volume $V_{o}=25\mu L$. The automaton grid comprises 200 × 200 square elements where each element can accommodate a cell with a 25μ*m* diameter. In this configuration the volume of one cell, which represents the intracellular volume in this case, is approximately $V_{i}=8pL$.

#### Transport parameters

The transport parameters of TMZ through the tumor cell membrane are available for the in vitro experiments where $V_{i}^{\;\;\;pop\;}$ was representing the intracellular volume for the entire cell population (see above). In the cellular automaton, each cell is considered individually so the intracellular volume $V_{i}$ now concerns a single cell. The influx and efflux parameters $p_{T}$ and $p_{T2}$ are rescaled according to the volume as follows:

(A1) $$\frac{p_{T}}{V_{i}}=\frac{p_{T}^{\;\;\;\mathrm{pop}\;}}{V_{i}^{\;\;\;\mathrm{pop}\;}}\;\text{and}\;\frac{p_{T2}}{V_{i}}=\frac{p_{T2}^{\;\;\;\mathrm{pop}\;}}{V_{i}^{\;\;\;\mathrm{pop}\;}}$$

The parameters for the cell population are given in Ballesta et al.,^4^ The transport parameters across the membrane of one single tumor cells are then:

(A2) $$p_{T}=\frac{p_{T}^{\;\;\;\mathrm{pop}\;}}{V_{i}^{\;\;\;\mathrm{pop}\;}}\cdot V_{i}=\frac{0.0038}{7.10^{-6}}\times 8.10^{-12}=4.34.10^{-9}$$

(A3) $$p_{T2}=\frac{p_{T2}^{\;\;\;\mathrm{pop}\;}}{V_{i}^{\;\;\;\mathrm{pop}\;}}\cdot V_{i}=\frac{0.008}{7.10^{-6}}\times 8.10^{-12}=9.14.10^{-9}$$

## APPENDIX B

### MODEL VALIDATION WITH PK RESULTS FROM CELL CULTURE

PK results of Ballesta et al.,^4^ were reproduced with the hybrid model formulation. These results (Figure 1B) correspond to experimental measurements made on U87 cultured cells where the extracellular pH was monitored and prescribed as an input function. From this preliminary simulation we note that:

1. the intracellular PK for TMZ, MTIC, Cations and DNA adducts are strictly the same for all cells of the automaton (no variations are observed depending on the cell location in the 2D tumor);
2. changing the amount of cells (ie, the level of confluence in the automaton) does not produce any effects on the extracellular TMZ concentration which remains spatially homogeneous. This is not due to diffusion since the TMZ profile remains homogeneous in the absence of diffusion (ie, for D=0). The deactivation of exchanges between extracellular medium and cells does not modify the extracellular TMZ PK either (this latter observation is confirmed with the original ODE model).

From this we conclude that only the extracellular degradation (transformation) of TMZ matters for the extracellular TMZ PK (exchanges with the cells do not have any influence on this since the extracellular volume $V_{o}$ is large). Since TMZ degradation (transformation) is spatially homogeneous, diffusion does not play any role in this simulation that reproduces the particular context of an “in vitro setting” where there is no source of heterogeneity in the extracellular culture medium that provides an unlimited access to resources including oxygen, nutrients, and drugs.

## APPENDIX C

### COMPUTATION OF THE TUMOR OXYGENATION

The oxygen concentration can be computed using the cellular automaton grid as in^26^, ^27^ where full details can be found. To sum up, the simulation domain that represents the tissue is assumed to be homogeneously paved with capillaries from which oxygen is delivered depending on the oxygen concentration locally available $O\left(x,y,t\right)$ and the vessels permeability $\gamma_{p}$. The oxygen then diffuses in the tissue and is consumed by the tumor cells that occupy the automaton grid at positions $\left(i,j\right)$ with uptake rate α. The resulting equations for the spatiotemporal evolution of oxygen is given by:

(C1) $$\frac{\partial O}{\partial t}=D_{o}\nabla^{2}O+\gamma_{p}v_{i,\;j}\left(O_{v}-O\right)-\alpha c_{i,j}$$

where $D_{o}$ is the oxygen diffusion coefficient, $O_{v}$ is the intravascular oxygen concentration and $v_{i,j}$ represents the vascular component given by:

(C2) $$v_{i,j}=1-c_{i,j}$$

meaning that in the presence of tumor cells, the capillaries are locally degraded (ie, the vascular map appears as the reversed image of the tumor cells map). Oxygen diffusion (equation A4) is simulated until a stationary state is reached.

## APPENDIX D

### COMPUTATION OF TMZ INTRACELLULAR STEADY STATE CONCENTRATION IN A CELL POPULATION

The steady state of TMZ intracellular concentration$T_{i}^{*}$ in a single cell can easily be derived from equations ((1) and (2)), and is equal to:

(D1) $$T_{i}^{*}\left(p_{T},p_{T2}\right)=\frac{p_{T}T_{\mathrm{tot}}}{p_{T}+p_{T2}}$$

where $p_{T}$ and $p_{T2}$ are the rate constants of TMZ cellular uptake and efflux, respectively.

We then computed TMZ cumulative intracellular steady state level in an heterogeneous cell population in which each cell displayed a different value of $p_{T}$, respectively $p_{T2}$. The studied intervals were here $\left[a_{T},b_{T}]=[0.2p_{T}^{†},1.8p_{T}^{†}\right]$ and $\left[a_{T2},b_{T2}]=[0.2p_{T2}^{†},1.8p_{T2}^{†}\right]$, respectively, which corresponded to a deviation of 80% of the value of $p_{T}^{†}$ or $p_{T2}^{†}$ estimated from experimental data in U87 cells.

TMZ cumulative steady state level was then computed by integrating $T_{i}^{*}$ with respect to $p_{T}$ (denoted $I_{1}$), respectively $p_{T2}$ (denoted $I_{2}$) on the studied interval. This is equivalent to model an infinity of cells whose parameters are uniformly selected in the predefined intervals. The same quantities were computed for a homogeneous cell population with no inter‐cell variability, that is, in which $p_{T}=p_{T}^{†}$ and $p_{T2}=p_{T2}^{†}$ for all cells (denoted $I_{0}^{T}$ and $I_{0}^{T2}$). The following formulas were derived:

(D2) $$I_{0}^{T}\left(p_{T}^{†},p_{T2}^{†}\right)=\int_{a_{T}}^{b_{T}}\frac{p_{T}^{†}T_{\mathrm{tot}}}{p_{T}^{†}+p_{T2}^{†}}dp_{T}=\left(b_{T}-a_{T}\right)\frac{p_{T}^{†}T_{\mathrm{tot}}}{p_{T}^{†}+p_{T2}^{†}}$$

(D3) $$I_{1}\left(p_{T2}^{†}\right)=\int_{a_{T}}^{b_{T}}\frac{p_{T}T_{\mathrm{tot}}}{p_{T}+p_{T2}^{†}}dp_{T}=T_{\mathrm{tot}}\left(b_{T}-a_{T}-\text{ln}\left(\frac{p_{T2}^{†}+b_{T}}{p_{T2}^{†}+a_{T}}\right)\right)$$

(D4) $$I_{0}^{T2}\left(p_{T}^{†},p_{T2}^{†}\right)=\int_{a_{T2}}^{b_{T2}}\frac{p_{T}^{†}T_{\mathrm{tot}}}{p_{T}^{†}+p_{T2}^{†}}dp_{T2}=\left(b_{T2}-a_{T2}\right)\frac{p_{T}^{†}T_{\mathrm{tot}}}{p_{T}^{†}+p_{T2}^{†}}$$

(D5) $$I_{2}^{T}\left(p_{T}^{†}\right)=\int_{a_{T2}}^{b_{T2}}\frac{p_{T}^{†}T_{\mathrm{tot}}}{p_{T}^{†}+p_{T2}^{}}dp_{T2}=T_{\mathrm{tot}}p_{T}^{†}\text{ln}\left(\frac{p_{T}^{†}+b_{T}}{p_{T}^{†}+a_{T}}\right)$$

## References

[1] Stupp R , Mason WP , van den Bent MJ , et al. Radiotherapy plus concomitant and adjuvant temozolomide for glioblastoma. N Engl J Med. 2005;352:987‐996. 15758009 10.1056/NEJMoa043330

[2] Niewald M , Berdel C , Fleckenstein J , Licht N , Ketter R , Rube C . Toxicity after radiochemotherapy for glioblastoma using temozolomide–a retrospective evaluation. Radiat Oncol. 2011;6:141. 22017800 10.1186/1748-717X-6-141 PMC3213071

[3] Syro LV , Rotondo F , Camargo M , Ortiz LD , Serna CA , Kovacs K . Temozolomide and Pituitary tumours: current Understanding, Unresolved Issues, and Future Directions. Front Endocrinol (Lausanne). 2018;15(9):318. 10.3389/fendo.2018.00318 PMC6013558 29963012

[4] Ballesta A , Zhou Q , Zhang X , LV H , Gallo JM . Multiscale design of cell‐type‐specific pharmacokinetic/pharmacodynamic models for personalized medicine: application to temozolomide in brain tumours. CPT Pharmacometrics Syst Pharmacol 2014;3:e112. 24785551 10.1038/psp.2014.9 PMC4017092

[5] Thomas A , Tanaka M , Trepel J , Reinhold WC , Rajapakse VN , Pommier Y . Temozolomide in the Era of Precision Medicine. Cancer Res. 2017;77(4):823‐826. 28159862 10.1158/0008-5472.CAN-16-2983 PMC5313339

[6] Denny BJ , Wheelhouse RT , Stevens MFG , Tsang LLH , Slack JA . NMR and molecular modeling investigation of the mechanism of activation of the antitumor drug temozolomide and its interaction with DNA. Biochem. 1994;33:9045‐9051. 8049205 10.1021/bi00197a003

[7] Gatenby RA , Gawlinski ET , Gmitro AF , Kaylor B , Gillies RJ . Acid‐mediated tumour invasion: a multidisciplinary study. Cancer Res. 2006;66(10):5216‐5223. 16707446 10.1158/0008-5472.CAN-05-4193

[8] Huber V , Camisaschi C , Berzi A , et al. Cancer acidity: an ultimate frontier of tumour immune escape and a novel target of immunomodulation. Semin Cancer Biol. 2017;43:74‐89. 28267587 10.1016/j.semcancer.2017.03.001

[9] Peppicelli S , Andreucci E , Ruzzolini J , et al. The acidic microenvironment as a possible niche of dormant tumour cells. Cell Mol Life Sci. 2017;74(15):2761‐2771. 28331999 10.1007/s00018-017-2496-y PMC11107711

[10] Fais S , Venturi G , Gatenby B . Microenvironmental acidosis in carcinogenesis and metastases: new strategies in prevention and therapy. Cancer Metastasis Rev. 2014;33(4):1095‐1108. 25376898 10.1007/s10555-014-9531-3 PMC4244550

[11] Hegde M , Roscoe J , Cala P , Gorin F . Amiloride kills malignant glioma cells independent of its inhibition of the sodium‐hydrogen exchanger. J Pharmacol Exp Ther. 2004;310:67‐74. 15010500 10.1124/jpet.103.065029

[12] Gatenby RA , Gawlinski ET . A reaction‐diffusion model of cancer invasion. Cancer Res. 1996;56:5745‐5753. 8971186

[13] Webb SD , Sherratt JA , Fish RG . Mathematical Modelling of Tumour Acidity: regulation of Intracellular pH. J Theor Biol. 1999;196:237‐250. 9990741 10.1006/jtbi.1998.0836

[14] Al‐Husari M , Webb SD . Regulation of tumour intracellular pH: a mathematical model examining the interplay between H+ and lactate. J Theor Biol. 2013;322:58‐71. 23340437 10.1016/j.jtbi.2013.01.007

[15] McGillen JB , Gaffney EA , Martin NK , Maini PK . A general reaction‐diffusion model of acidity in cancer invasion. J Math Biol. 2014;68:1199‐1224. 23536240 10.1007/s00285-013-0665-7

[16] Smallbone K , Gatenby RA , Maini PK . Mathematical modelling of tumour acidity. J Theor Biol. 2008;255:106‐112. 18725231 10.1016/j.jtbi.2008.08.002

[17] Al‐Husari M , Murdoch C , Webb SD . A cellular automaton model examining the effects of oxygen, hydrogen ions and lactate on early tumour growth. J Math Biol. 2014;69(4):839‐873. 23982261 10.1007/s00285-013-0719-x

[18] Powathil GG , Gordon KE , Hill LA , Chaplain MAJ . Modelling the effects of cell‐cycle heterogeneity on the response of a solid tumour to chemotherapy: biological insights from a hybrid multiscale cellular automaton model. J Theor Biol. 2012;308:1‐19. 22659352 10.1016/j.jtbi.2012.05.015

[19] Stéphanou A , Volpert V . Hybrid modelling in biology: a classification review. Math Mod Nat Phenom. 2016;11(1):37‐48.

[20] Lopes IC , de Oliveira SCB , Oliveira‐Brett AM . Temozolomide chemical degradation to 5‐aminoimidazole‐4‐carboxamide ‐ Electromechanical study. J Electroanal Chem. 2013;704:183‐189.

[21] Pavlova NN , Thompson CB . The Emerging hallmarks of cancer metabolism. Cell Metab. 2016;23(1):27‐47. 26771115 10.1016/j.cmet.2015.12.006 PMC4715268

[22] Rohren EM , Turkington TG , Coleman RE . Clinical applications of PET in oncology. Radiology. 2004;231(2):305‐332. 15044750 10.1148/radiol.2312021185

[23] Verger A , Langen KJ . PET Imaging in glioblastoma: use in clinical practice In: De Vleeschouwer S, editor. Glioblastoma. Brisbane (AU):Codon Publications; 2017. Chapter 9. 29251869

[24] Warburg O . The metabolism of tumours. London: Constable Press; 1930.

[25] Helmlinger G , Yuan F , Dellian M , Jain RK . Interstitial pH and pO2 gradients in solid tumours in vivo: high‐resolution measurements reveal a lack of correlation. Nat Med. 1997;3:177‐182. 9018236 10.1038/nm0297-177

[26] Caraguel F , Lesart AC , Estève F , Van der Sanden B , Stéphanou A . Towards the design of a patient‐specific virtual tumour. Comp Math Meth Med 2016;2016:7851789. 10.1155/2016/7851789 PMC5206790 28096895

[27] Stéphanou A , Lesart AC , Deverchère J , Juhem A , Popov A , Estève F . How tumour‐induced vascular changes alter angiogenesis: insights from a computational model. J Theor Biol. 2017;419:211‐226. 28223171 10.1016/j.jtbi.2017.02.018

[28] Estrella V , Chen T , Lloyd M , et al. Acidity generated by the tumour microenvironment drives local invasion. Cancer Res. 2013;73(5):1524‐1535. 23288510 10.1158/0008-5472.CAN-12-2796 PMC3594450

[29] Hashim AI , Zhang X , Wojtkowiak JW , Martinez GV , Gillies RJ . Imaging pH and metastasis. NMR Biomed. 2011;24:582‐591. 21387439 10.1002/nbm.1644 PMC3740268

[30] Damaghi M , Wojtkowiak JW , Gillies RJ . pH sensing and regulation in cancer. Front Physiol. 2013;4:370. 24381558 10.3389/fphys.2013.00370 PMC3865727

[31] Parks SK , Chiche J , Pouyssegur J . pH control mechanisms of tumour survival and growth. J Cell Physiol. 2011;226(2):299‐308. 20857482 10.1002/jcp.22400

[32] Kondo A , Yamamoto S , Nakaki R , et al. Extracellular Acidic pH Activates the Sterol Regulatory Element‐Binding Protein 2 to Promote tumour Progression. Cell Rep. 2017;18:2228‐2242. 28249167 10.1016/j.celrep.2017.02.006

[33] L'Allemain G , Paris S , Pouysségur J . Growth factor action and intracellular pH regulation in fibroblasts. J Biol Chem. 1984;259(9):5809‐5815. 6325450

[34] Song CW , Griffin R , Park HJ . Influence of tumour pH on therapeutic response In Teicher BA, eds. Cancer Drug Discovery and Development: cancer Drug Resistance. Totowa, NJ: Humana Press;2006:21‐42.

[35] Arnold JB , Kraig RP , Rottenberg DA . In Vivo Measurement of Regional Brain and tumour pH Using [14C]Dimethyloxazolidinedione and Quantitative Autoradiography. II: characterization of the Extracellular Fluid Compartment Using pH‐Sensitive Microelectrodes and [14C]Sucrose. J Cereb Blood Flow Metab 1986;6(4):435‐440. 3733903 10.1038/jcbfm.1986.76 PMC3047405

[36] McLean LA , Roscoe J , Jorgensen NK , Gorin FA , Cala PM . Malignant gliomas display altered pH regulation by NHE1 compared with nontransformed astrocytes. Am J Physiol Cell Physiol. 2000;278:C676‐C688. 10751317 10.1152/ajpcell.2000.278.4.C676

[37] Reshkin SJ , Bellizzi A , Caldeira S , et al. Na+/H+ exchanger‐dependent intracellular alkalinization is an early event in malignant transformation and plays an essential role in the development of subsequent transformation associated phenotypes. FASEB J. 2000;14(14):2185‐2197. 11053239 10.1096/fj.00-0029com

[38] White KA , Grillo‐Hill BK , Barber DL . Cancer cell behaviors mediated by dysregulated pH dynamics at a glance. J Cell Sci. 2017;130:663‐669. 28202602 10.1242/jcs.195297 PMC5339414

[39] Madshus IH . Regulation of intracellular pH in eukaryotic cells. Biochem J. 1988;250:1‐8. 2965576 10.1042/bj2500001 PMC1148806

[40] Curtis MJ , Alexander S , Cirino G , et al. Experimental design and analysis and their reporting I I: updated and sampled guidance for authors and peer reviewers. Br J Pharmacol. 2018;175:987‐993. 29520785 10.1111/bph.14153 PMC5843711

[41] Powathil GG , Swat M , Chaplain MAJ . Systems oncology: towards patient‐specific treatment regimes informed by multiscale mathematical modelling. Semin Cancer Biol. 2015;30:13‐20. 24607841 10.1016/j.semcancer.2014.02.003

[42] Stéphanou A , Fanchon E , Innominato P , Ballesta A . Systems Biology, Systems Medicine, Systems Pharmacology: the What and The Why. Acta Biotheor. 2018;66:345‐365. 10.1007/s10441-018-9330-2. 29744615

